# Symptomatic hyponatremia induced by low-dose cyclophosphamide in patient with systemic lupus erythematosus: A case report

**DOI:** 10.1097/MD.0000000000022498

**Published:** 2020-11-25

**Authors:** Jiali Chen, Yuebo Jin, Chun Li, Zhanguo Li

**Affiliations:** Department of Rheumatology & Immunology, Peking University People's Hospital, Beijing, China.

**Keywords:** cyclophosphamide, hyponatremia, systemic lupus erythematosus, syndrome of inappropriate antidiuretic hormone secretion

## Abstract

**Rationale:**

Cyclophosphamide (CY) is an alkylating agent used widely to treat cancer and autoimmune diseases. Hyponatremia is a common adverse effect of high-dose and moderate-dose of intravenous CY, but is rare in patients treated with low-dose (<15 mg/kg).

**Patient concerns:**

A 52-year-old woman with new-onset systemic lupus erythematosus (SLE) was treated with low-dose cyclophosphamide (8 mg/kg, CY), but showed sudden headaches, disorientation and weakness. Laboratory examinations revealed severe isovolumic hyponatremia along with low-serum osmolality and high urine osmolality.

**Diagnosis:**

The acute hyponatremia was consistent with the syndrome of inappropriate antidiuretic hormone secretion (SIADH) and was an adverse event of low-dose CY, with no evidence of endocrine, cancer, pulmonary, or cerebral abnormalities relevant to the SIADH.

**Intervention:**

The hyponatremia was resolved after the supplementation of NaCl solution.

**Outcomes:**

The hyponatremia was resolved without any complications.

**Lessons:**

Hyponatremia induced by low-dose CY should be recognized as an underlying life-threatening complication in clinical practice.

## 1 Introduction

Cyclophosphamide (CY) is an alkylating agent synthesized over 50 years ago, which is used widely in the treatment of malignancy and autoimmune diseases. Its well-known adverse effects include nausea, vomiting, alopecia, bone marrow suppression, infection, hemorrhagic cystitis, sterility, and malignancy.^[1]^ Hyponatremia is rarely been seen in patients treated with low-dose (<15 mg/kg) of intravenous CY therapy,^[1–3]^ although previous studies have been reported with the treatment of high (30–40 mg/kg)^[4,5]^ and moderate (20–30 mg/kg) doses.^[6,7]^ Though low-dose CY is being used widely in the treatment of rheumatic diseases, the rare adverse effect of hyponatremia is not extensively recognized in the rheumatology literature and we found only 5 cases are associated with rheumatic diseases. In this article, we report a patient with systemic lupus erythematosus (SLE) who suffered from recurrent hyponatremia following the administrations of low-dose CY. We also performed an extensive literature review of water intoxication or hyponatremia cases among patients treated with low-dose CY and analyzed the clinical characteristics of these patients.

## 2 Case report

**Figure 1:**
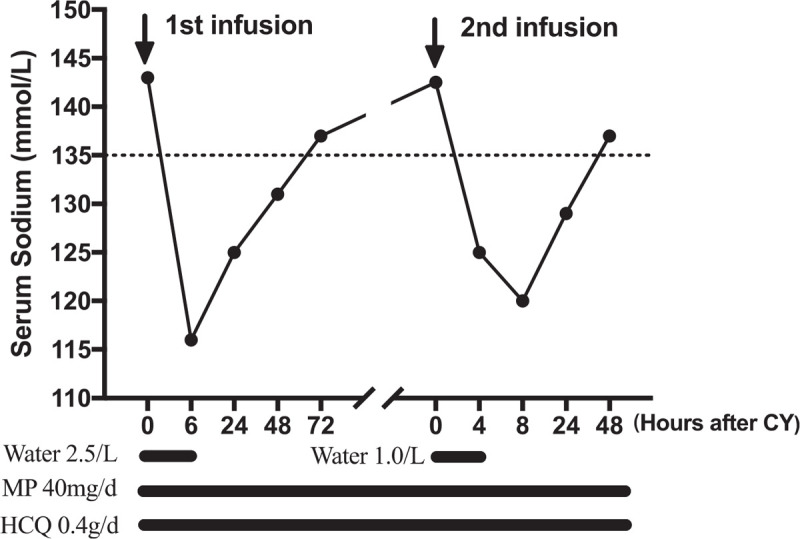
Time trend of serum sodium following the administration of low-dose cyclophosphamide. HCQ = hydroxychloroquine; MP = methylprednisolone; Pred = prednisolone.

A 52-year-old woman presented to our hospital in June 27, 2018, with one month history of oral ulcers, malar rash. Physical examination revealed vasculitis in her fingertips. Her leukocyte count was 2100/μL, lymphocyte proportion was 25.4%, and hemoglobin level was 98 g/L with positive Coombs test. Serum creatinine was normal and urinalysis showed hematuria and proteinuria with protein excretion of 1.32 g per day. Antinuclear antibody, anti-double-stranded DNA, anti-Sm, and anti-Ribonucleoprotein antibody were all positive, combined with a decreased level of complement. She met the American College of Rheumatology classification criteria for SLE and received methylprednisolone at 40 mg/day plus hydroxychloroquine (0.4 g/day). A week later, she was given the first administration of CY 400 mg (8 mg/kg) and was well-tolerated. Six hours later, she felt unwell with headaches, and then disorientation and weakness were gradually developed. Her blood pressure was 124/68 mmHg and emergency biochemistry examinations showed a significant decrease of serum sodium from 143 to 116 mmol/L. The serum osmolality was 253 mOsm per kilogram of water, while the urinary osmolality 506 mOsm per kilogram of water, and the urinary sodium 80 mmol/L. Her computed tomography scan of brain was normal, and cerebrospinal fluid examination excluded infection. Because her hyponatremia was acute and symptomatic, she was treated with intravenous infusion of 3% NaCl solution at a rate of 30 mL/h for 8 hours and 0.9% NaCl at a rate of 75 ml/L for 14 hours, accompanied with water restriction. Her serum sodium slowly rose to 125 mmol/L after 24 hours and 131 mmol/L after 48 hours, and her mental status recovered. The patient made a complete recovery and was discharged on July 10, 2018, when the serum sodium reached 142 mmol/L (Fig. 1).

Given that 2.5 L water were rapidly ingested in 6 hours after the first infusion of CY which may attribute to the hyponatremia, the second infusion of CY was used under direct supervision with the patient's permission. She was admitted on July 16, 2018, and her serum sodium was 142.5 mmol/L before the infusion of CY. She drunk about 1.0 L of fluid and her serum sodium dropped to 125 mmol/L 4 hours later. The serum osmolality was 268 mOsm per kilogram of water, while the urinary osmolality 537 mOsm per kilogram of water, and the urinary sodium 64 mmol/L. Additionally, serum sodium continually decreased to 120 mmol/L though she was treated with 0.9% NaCl and fluid restriction. In that case, oral sodium chloride was added and serum sodium levels gradually improved to 129 mmol/L 24 hours later and 137 mmol/L after 48 hours (Fig. 1).

The patient had isovolumic hyponatremia with low-serum osmolality and high urine osmolality, which was consistent with syndrome of inappropriate antidiuretic hormone secretion (SIADH). We suspected CY as the etiology of hyponatremia for no other cause was definite. Her renal function and serum electrolyte were normal, with no evidence of endocrine, cancer, pulmonary or cerebral abnormalities, and another suspicious drug relevant to the SIADH. Furthermore, she suffered from similar manifestations in the second administration of CY. Eventually, she was discharged on July 23, 2018, with oral medication of prednisolone 50 mg/day, MMF 1000 mg/day, and hydroxychloroquine 400 mg/day but without any further plans of intravenous CY. This case study was approved by the ethics committee of Peking University People's Hospital, and the patients provided informed consent.

## 3 Discussion

Hyponatremia is the most common body fluid and electrolyte disorder in clinical practice, which is defined as an excess of water in the extracellular fluid and serum sodium<135 mmol/L.^[8]^ The syndrome of inappropriate secretion of antidiuretic hormone (SIADH) is the most prevalent cause of hyponatremia.^[9]^ SIADH was first reported in patients of bronchogenic carcinoma with inappropriate secretion of the arginine vasopressin (AVP).^[10]^ The secretion of AVP is independent of plasma osmolality and is one of the most important pathogenesis of patients with SIADH. Because not all patients with SIADH have increased serum levels of AVP, the term was inaccurate for this condition and syndrome of inappropriate antidiuresis (SIAD) was proposed as an accurate term.^[11]^ Various etiologies can cause SIADH, and they can be categorized as related to malignant diseases, pulmonary diseases, infection, and disorders of the central nervous system. Additionally, several drugs, especially chlorpropamide, tricyclic antidepressants, and several cytotoxic drugs including vincristine, cyclophosphamide can stimulate the release of arginine vasopressin or enhance its action, and they could be served as causes of SIADH.^[9]^

Severe hyponatremia has been reported in patients treated with high-dose (30–40 mg/kg) and moderate-dose (20–30 mg/kg),^[4–7]^ while life-threatening acute hyponatremia is rare in low-dose CY. As far, a total of 14 cases reports have been described following low-dose CY therapy (<15 mg/kg). The data is summarized in Table 1. Seven cases are associated with autoimmune diseases (AIDs), including SLE,^[1–3]^ Sjögren syndrome,^[12]^ pemphigus,^[13]^ systemic sclerosis,^[14]^ and rapidly histological glomerulonephritis.^[15]^ The other 7 cases are about malignancy associated with multiple myeloma,^[7,16]^ breast cancer.^[17–21]^ Most of them are female except 2 men, and the average age was 56 years (range 26–69 years). These patients had active disease activity and combined therapies included corticosteroids, hydroxychloroquine, and some cytotoxic drugs including azathioprine, thalidomide, vincristine, fluorouracil, epirubicin, docetaxel, and vinorelbine. Dose of CY ranged from 500 mg to 15 mg/kg per infusion. Hyponatremia occurs 5 to 12 hours after the administration of CY with fluid intake estimated 1 to 3 L. Serum sodium was changed from 136 (range 129–138) mmol/L to 116 (106–124) mmol/L in average, accompanied with the symptoms of nausea, vomiting, seizure, weakness, and other neurological symptoms related to hyponatremia.

**Table 1:** Overview of reported cases of hyponatremia or SIADH associated with low-dose cyclophosphamide.

Ref.	References	Patients (age/sex)	Disease	Dose of CY used	Fluid intake (estimated, L)	Time after CY, h	Sodium before CY, mmol/L	Sodium after CY, mmol/L	Combined drug (s)
^[3]^	McCarron, 1995	59/Female	SLE	10 mg/kg	2.5	12	138	116	Pred 20 mg/d
^[1]^	Salido M, 2003	48/Female	SLE	12.5mg/kg	3	8	–	119	Pred 1 mg/kg/d AZA 3mg/kg/d
	Salio M, 2003	53/Female	SLE, SS	500 mg	3	7	NA	119	Pred 50 mg
^[2]^	Koo TY, 2007	27/Female	Lupus nephritis	14.8 mg/kg	2	5	135	114	Pred 50 mg HCQ 200 mg
^[12]^	Spital, 1997	NA	SS	<15 mg/kg	NA	NA	NA	117	NA
^[13]^	Jayachandran NV, 2009	49/Female	Systemic sclerosis	500 mg	NA	24	NA	106	Pred 20 mg/d
^[14]^	Karthika Nataraian, 2009	26/Female	Pemphigus	<15 mg/kg	NA	NA	NA	117	Pred
^[15]^	Esposito P, 2017	56/Male	RPGN	8 mg/kg	2	48	137	122	Pred 50 mg/d
^[16]^	Webberley M.J, 1989	68/Male	Multiple Myeloma	500 mg	3	48	138	108	NA
^[17]^	Hwang SB, 2011	56/Female	Breast cancer	600 mg/m^2^	1	50	NA	116	DOX 90 mg, DXM
^[18]^	Bruining DM, 2011	64/Female	Breast cancer	500 mg/m^2^	1.5–2.0	28	134	107	5*-*FLU 500 mg/m^2^ EPI, 100 mg/m2
^[7]^	Gilbar PJ, 2012	69/Female	Multiple Myeloma	8 mg/kg	1.5	48	129	113	Thali, 100 mg/d DXM 40mg/d
^[19]^	Geng C, 2014	54/Female	Breast cancer	500 mg/m^2^	2	13	136	120	DXM 10 mg
	Geng C, 2014	58/Female	Breast cancer	500 mg/m^2^	2	NA	NA	124	EPI 100 mg/m^2^
^[20]^	Michelle Baker, 2014	58/Female	Breast cancer	15 mg/kg	NA	48–72	NA	117	DXM 12 mg DTX 130 mg.
	Michelle Baker, 2014	56/Female	Breast cancer	15 mg/kg	NA	24	NA	113	DOX 95 mg
^[21]^	Shereen Elazzazy, 2014	43/Female	Breast cancer	600 mg/m^2^	NA	72	138	112	Chlorthalidone 12.5 mg

Reports of CY-induced hyponatremia or SIADH usually occurs 4 to 12 hours after the infusion of CY which may be related to the active alkylating metabolite of CY. The maximum antidiuretic effect of this metabolite occurs at 10 to 14 hours after drug administration though the half-life time of CY in serum is about 6 to 7 hours.^[22,23]^ The mechanisms of water intoxication and SIADH induced by cyclophosphamide have not been clearly understood, though several hypotheses have been proposed to explain the underlying mechanisms. CY or active form of CY can either stimulate AVP release or enhance its renal effects and cause hyponatremia finally. Harlow et al^[24]^ revealed the loss of Herrings bodies and degranulation of various hypothalamic neurosecretory organelles in 1 patient who had high-dose CY with the postmortem examination. These organelles can act indirectly by causing the inappropriate secretion of AVP, which has been demonstrated with ifosfamide, a similar structural analog to CY.^[25]^ However, Pratt et al^[26]^ indicated that serum ADH levels have not changed apparently after the administration of CY. Furthermore, DeFronzo et al^[27]^ believed that alkylating metabolite of CY had a direct effect on the kidney resulting in enhanced permeability of the distal tubules to water without altering the glomerular filtration rate or urine sodium excretion. Additionally, CY might cause hyponatremia by upregulating expression of ADH receptor V2R and aquaporin 2 through suppression of interleukin-1 and tumor necrosis factor-α, which act as negative regulators of VR2 expression.^[28]^

Fluid intake ranging from 2 to 3 L after the administration of CY to lower the risk of hemorrhagic cystitis is an important factor for water intoxication. Bruining *et al*.^[18]^ reported to have extreme amounts of fluids intake in a short time may cause severe hyponatremia. The routine uses of Mesna and ingesting 1 L of fluid above patients’ usual intake in the following 24 hours may reduce the risk of water intoxication.^[1]^

Caution is necessary in patients with significant renal disorders because renal failure or hypoalbuminemia could prolong the half-life of CY or its alkylating metabolites and deteriorate the water-retention.^[1]^ Besides, simultaneous administration of corticosteroid, non-steroid anti-inflammatory drugs, antidepressants, and other cytotoxic drugs may accelerate the acute water intoxication.^[15]^

In conclusion, the aforementioned case highlights the rare complication of water intoxication after the administration of low-dose CY. Our case, together with other reported cases, emphasizes SIADH indeed should be kept in mind in clinical practice when intravenous cyclophosphamide is applied, especially in patients with other underlying risk factors.

## Author contributions

**Conceptualization:** Jiali Chen, Chun Li.

**Formal analysis:** Jiali Chen.

**Investigation:** Jiali Chen, Chun Li.

**Supervision:** Yuebo Jin, Chun Li, Zhanguo Li.

**Writing – original draft:** Jiali Chen.

**Writing – review & editing:** Jiali Chen, Yuebo Jin, Chun Li, Zhanguo Li.
